# Increasing loneliness in Japan, 1983–2023: a cross-temporal meta-analysis

**DOI:** 10.3389/fpsyg.2026.1824941

**Published:** 2026-04-14

**Authors:** Momo Homma, Kenkichi Takase

**Affiliations:** Department of Psychology, Chuo University, Tokyo, Japan

**Keywords:** Japan, loneliness, mental health, meta-analysis, UCLA Loneliness Scale

## Abstract

Loneliness and social isolation have become crucial in Japan, but research on how loneliness has changed over time is lacking. This study conducted a cross-temporal meta-analysis to address this gap. Studies were identified from PubMed, Web of Science, J-Stage, and CiNii; exclusion criteria were missing means, absence of the University of California, Los Angeles (UCLA) Loneliness Scale, unclear year of study, duplicate data, and lack of sample size or clear scale use. We identified an overall increase in loneliness between 1983 and 2023, particularly among adolescents. Regression analysis indicated that developmental stage, number of scale items, and type of Japanese translation of the UCLA Loneliness Scale were related to loneliness trends. While male participants consistently reported higher loneliness, results suggested a more significant increase among female participants. Some social indicators were associated with changes in loneliness. Despite various contributing factors, the underlying causes remain unclear, highlighting the need for further research.

## 1 Introduction

Against the backdrop of significant changes in Japan’s social environment, loneliness and social isolation have become urgent concerns in Japanese society. Japan has been experiencing an increasing number of single-person households and a shift toward nuclear families, alongside a declining birthrate and aging population [Ministry of Health, Labour and Welfare (MHLW), 2022a; Ministry of Health, Labour and Welfare (MHLW), n.d.]. Additionally, traditional lifetime employment is undergoing a transition to part-time or contingent employment because of rapid globalization (Statistics Bureau of Japan, 2025). Moreover, the spread of the Internet has changed individuals’ lifestyles [Ministry of Internal Affairs and Communications (MIC), 2019]. Because of the changes in the social environment, “social connections” are progressively fading from society.

The global COVID-19 pandemic has worsened the situation. To prevent the spread of infection, the Japanese government urged the avoidance of the three Cs: “closed spaces with poor ventilation,” “crowded places with many people nearby,” and “close contact settings such as close-range conversations” [Ministry of Health, Labour and Welfare (MHLW), 2020]. To ensure safety, people had to alter their routines, shifting quickly to online communication as in-person interactions were limited. These changes led to increased loneliness and social isolation, which are now pressing challenges in Japanese society.

“Loneliness” is defined as a discrepancy between achieved and “needed” levels of social contact (Peplau and Perlman, 1979). “Social isolation” is defined as a tangible absence of strong and supportive social networks (Taylor, 2020). Therefore, loneliness is a subjective emotional state, whereas social isolation is an objective condition. An individual can be lonely but not isolated, or isolated but not lonely. Moreover, loneliness and social isolation are only low to moderately correlated (Taylor et al., 2023). Among young individuals, no difference in time spent alone has been found between those who feel lonely and those who do not (Cacioppo et al., 2000).

Several studies have found associations between loneliness and various health risks, including associations with mental health risks, such as depression (Adams et al., 2004), suicidal thoughts (McClelland et al., 2020), anxiety (Steen et al., 2022), perceived stress (Wang et al., 2024), and substance use disorders (Ingram et al., 2020), as well as physical health risks such as cardiovascular diseases (Caspi et al., 2006), cancers (Kraav et al., 2021), cognitive function (Harrington et al., 2023), sleep disorders (Harris et al., 2013), and metabolic syndrome (Whisman, 2010). Additionally, loneliness has been associated with health behaviors, such as impaired control of eating behaviors (Hanna et al., 2023) and low physical activity levels (Pels and Kleinert, 2016). These health behaviors further increase morbidity and correlate with poor health outcomes. Moreover, a meta-analysis reported that lonely individuals had 26% greater odds of early mortality compared with non-lonely individuals (Holt-Lunstad et al., 2015). Thus, the alleviation of loneliness is a crucial issue for the national government.

Japan is the second country in the world, following the United Kingdom, to appoint a Minister of State for Measures for Loneliness and Isolation and is a global pioneer in addressing loneliness and social isolation. Further, the Act on the Advancement of Measures to Address Loneliness and Isolation stipulates promoting measures to address loneliness and isolation across all societal sectors as its basic principle (Cabinet Office, 2023). The national survey of loneliness and isolation is one of the Cabinet Office’s large-scale projects (Cabinet Office, 2024a); a nationwide survey of loneliness and isolation was conducted from 2021 to 2023 with 20,000 individuals over the age of 16 using random sampling, approximately half of whom answered the survey annually. Among them, in 2023, 4.8% individuals (5.3% men and 4.2% women) reported that they “always or often” felt lonely, with a higher tendency among those aged 20 to 50 years.

However, given the 3-year survey period, changes in loneliness prevalence and characteristics remain unclear. Moreover, no study has analyzed these potential changes in loneliness over time. If an increase in loneliness is revealed, associated variables can be examined to inform effective countermeasures. If no increase is revealed, the reasons behind a perceived increase can be examined, similarly informing the design of effective measures.

Previous studies investigated changes in loneliness over time using cross-temporal meta-analysis (Buecker et al., 2021; Clark et al., 2015), which is a meta-analytic technique that integrates the mean score of a variable by year of study and reveals changes over time (Twenge and Campbell, 2001). A past meta-analysis showed that loneliness increased globally from 1976 to 2019 (Buecker et al., 2021), while another showed a decrease among high school and university students in the United States from 1978 to 2009 (Clark et al., 2015). Thus, cross-temporal analysis has been widely used to assess changes over time in self-esteem (Oshio et al., 2014; Twenge and Campbell, 2001), attachment style (Konrath et al., 2014), and gender stereotypes (Eagly et al., 2020). Accordingly, cross-temporal meta-analysis was deemed well-suited for the present study.

In this study, the mean score of the University of California, Los Angeles (UCLA) Loneliness Scale was analyzed by investigation year (Russell, 1996; Russell et al., 1978, 1980). Loneliness began to be measured quantitatively in Japan after the Japanese version of the Revised UCLA Loneliness Scale was developed in 1983 (Kudoh and Nishikawa, 1983). The scale has since been translated and modified, developing versions with fewer items; thus, various types of the UCLA Loneliness Scale have emerged for various individuals and situations (Hughes et al., 2004; Masuda et al., 2012). Other scales, such as the Three-Item Loneliness Scale (Igarashi, 2019), have also been used to measure loneliness in Japan; however, only the UCLA Loneliness Scale has been used frequently enough to allow for secondary analysis. Changes in the mean score of the UCLA Loneliness Scale over time can represent changes in loneliness over time.

Using cross-temporal meta-analysis, the present study aimed to (a) reveal the changes in loneliness over time in Japanese society and (b) examine related factors. It hypothesized that loneliness is increasing in Japanese society, given that loneliness and social isolation have become major social issues in recent years.

## 2 Methods

### 2.1 Transparency and openness

We adhered to the PRISMA guidelines for meta-analytic reporting (Page et al., 2021). All meta-analytic data and codes are available in the Supplementary material. This study was not preregistered.

### 2.2 Literature search

Four databases were used to locate studies: PubMed, Web of Science, J-Stage, and CiNii. PubMed is a database of worldwide scientific and medical research. Web of Science is a global database of scholarly articles. J-STAGE is a database that provides access to various resources published in Japan. CiNii is a database of academic information published in Japan. To find studies that used the UCLA Loneliness Scale in Japan, English-language databases (PubMed and Web of Science) were searched using the keywords “UCLA loneliness scale, Japan” and “UCLA loneliness scale, Japanese” on February 4, 2024. Japanese databases (J-Stage and CiNii) were searched using the keyword “UCLA loneliness scale” (in Japanese) on February 4, 2024. To include as much as studies in the analysis, we used different Japanese translations and versions of the UCLA Loneliness Scale. To account for potential differences arising from these variations (e.g., number of items, point scale), we examined their effects using multiple regression analyses.

**Figure 1:**
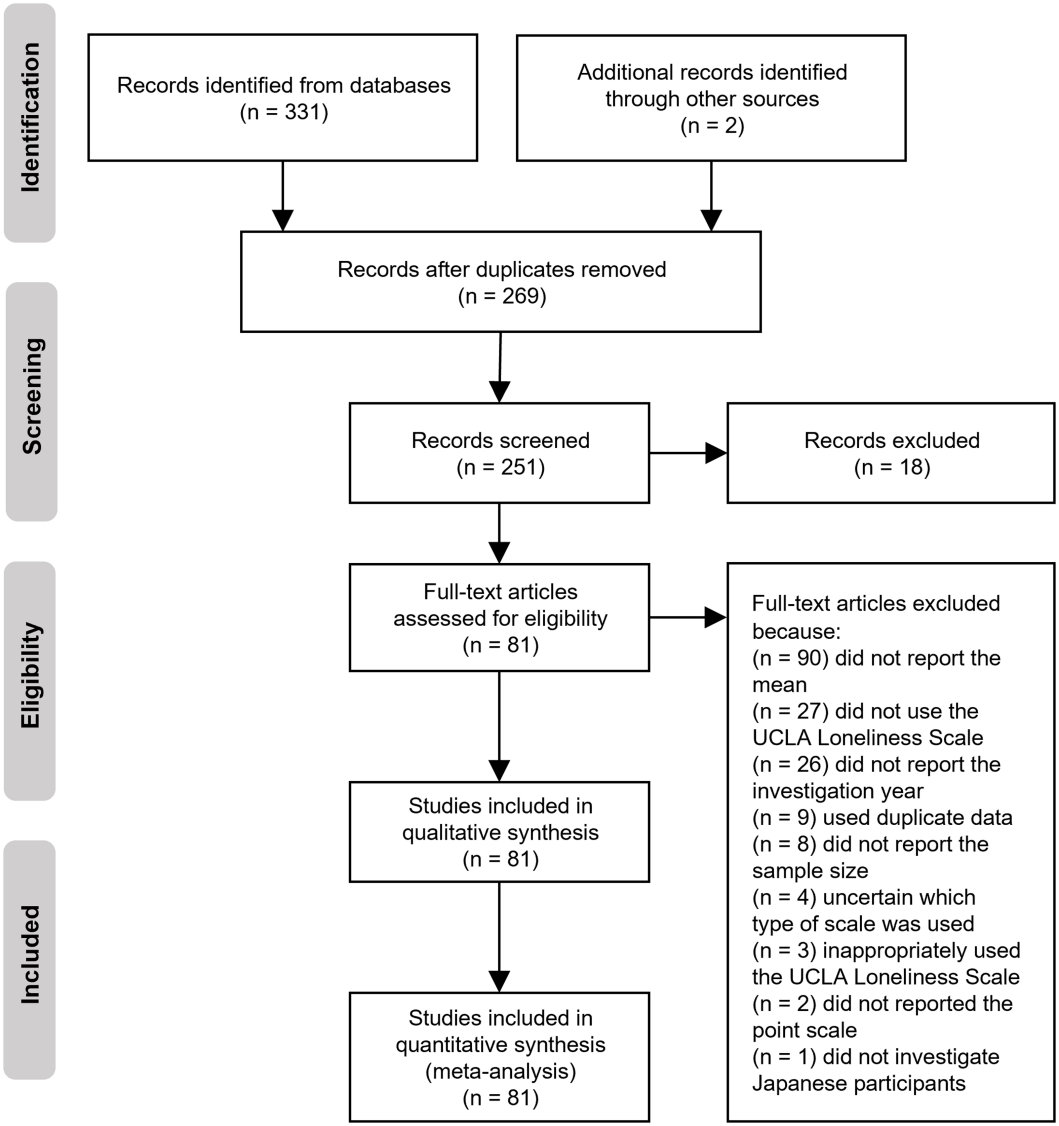
Flow chart of the selection process and the number of articles in each period.

Additionally, some studies were added after being discovered during the review of the initially retrieved articles. Two independent assessors, who also conducted the title and abstract reviews, applied the following inclusion criteria: (a) participants were Japanese; (b) participants’ UCLA loneliness scale scores were measured. The literature search identified 333 studies. After removing duplicates, the remaining studies were screened by reading their abstracts. Subsequently, 18 studies were excluded, and thus the full texts of 251 studies were assessed for eligibility. Ultimately, 81 studies were included in this meta-analysis after applying the exclusion criteria (Figure 1; Supplementary Table 1).

### 2.3 Types of analyses

After coding and adjusting the scores, statistical analyses were conducted using IBM SPSS Statistics Version 29.0.2.0 (IBM Corporation, 2023).

#### 2.3.1 Simple regression analysis

To perform simple regression analyses, three datasets that exceeded the mean score by 2 standard deviations were removed as outliers. Linear and quadratic regression analyses were performed to determine both linear and non-linear changes in loneliness.

#### 2.3.2 Multiple regression analysis

Multiple regression analyses were conducted using the same datasets used in the simple regression analyses. The weighted mean UCLA loneliness score was set as the response variable, and the point scale, number of items on the scale, type of Japanese translation of the scale, developmental stage, and investigation year were set as explanatory variables. Some variables were converted into dummy variables to conduct multiple regression analysis. Regarding point scales, 90.16% of the data used a 4-point scale; these were coded as 1, and all others were coded as 0. Regarding the number of items, 82.51% of the data used 20 items; these were coded as 1 and others as 0.

Regarding the type of Japanese translation, no single translation was dominant, and five patterns of dummy variables were coded (Kudo, Toyoshima, Masuda, and Arimoto; e.g., Kudo’s translation was coded as 1 and others as 0). Regarding developmental stages, data from adolescents were most prevalent. However, to determine the effect of developmental stages, dummy variables were created for adolescence, adulthood, and senium. Investigation years were centered by the mean.

#### 2.3.3 Sex difference analysis

First, the total effect size of sex was calculated, and a simple regression analysis was used to examine changes in effect sizes over time. Effect sizes were calculated from studies that reported the average loneliness score of male and female participants separately. The inverse variance method was chosen to weight the scores, as these data were not adjusted; thus, the inverse of the effect size’s variance was used as the weight.

Additionally, simple and multiple regression analyses were conducted for data on male and female participants. These analyses used adjusted scores, and the sample size method was chosen to weight the scores.

#### 2.3.4 Effect size analysis of the COVID-19 pandemic

Data from 2017 to 2019 were used as pre-pandemic data, and those from 2020 to 2022 were used as post-pandemic data. Because the adjusted scores could not be weighted using the inverse variance method, only data using the 4-point scale, 20 items, and scores starting from 1 were included.

### 2.4 Social indicators

According to Twenge and Campbell (2001), social indicators must be easily obtained and quantified as meaningful continuous variables; they must also be capable of representing general trends. We extracted data related to social connectedness, economic conditions, and overall threats. Social connectedness included the number of single-person households, average household size, marriage rate, divorce rate, and social interactions within the community [Cabinet Office, 2024b; Ministry of Health, Labour and Welfare (MHLW), 2023; Ministry of Health, Labour and Welfare (MHLW), 2025; Ministry of Internal Affairs and Communications (MIC), 2025a]. Economic conditions included GDP and the total unemployment rate [Cabinet Office, 2025; Ministry of Internal Affairs and Communications (MIC), 2025b]. Overall threats included the average time spent using the Internet and the percentage of Internet users [Ministry of Internal Affairs and Communications (MIC), 2025c].

We conducted a time-lag analysis to determine the correlation between social indicators and loneliness. We matched the social indicators with the adjusted loneliness scores of each study at five time points: 10 and 5 years prior to data collection, and 10 and 5 years after data collection.

## 3 Results

### 3.1 Simple regression analysis

#### 3.1.1 Overall

**Figure 2:**
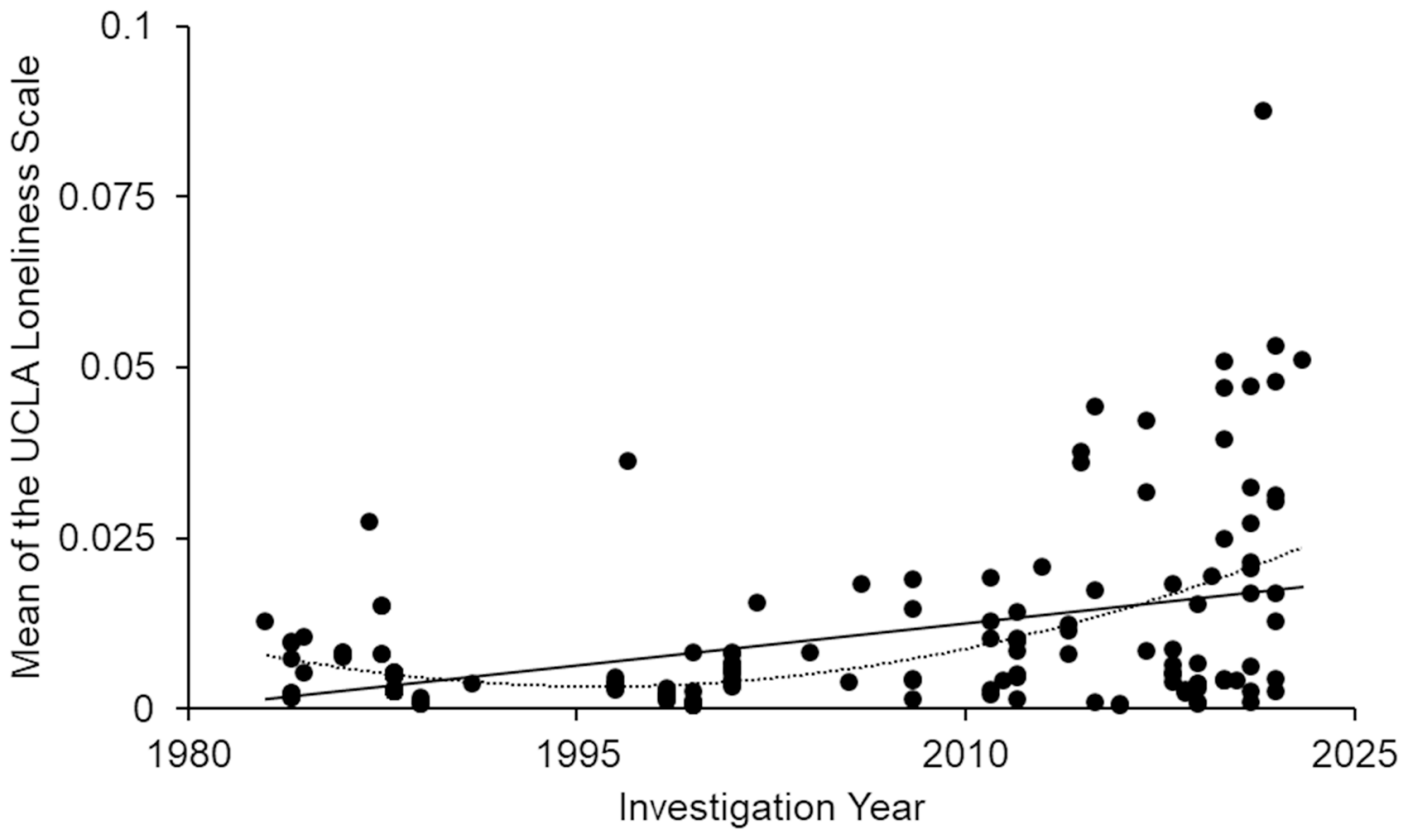
UCLA loneliness score changes over time in Japanese society; *d* = 183.

The linear regression analysis showed r = 0.428 (*p* < 0.001), denoting a low-to-moderate correlation between the mean UCLA loneliness score and investigation year (Figure 2). The quadratic regression results were also significant (r = 0.499, *p* < 0.001).

To examine the robustness of these findings, we conducted several additional analyses. First, when including outliers, the effects remained significant, although attenuated (linear: r = 0.205, *p* = 0.005; quadratic: r = 0.251, *p* = 0.003). Second, analyses using a homogeneous subset of studies (20 items, 4-point scale) yielded consistent results (linear: r = 0.356, *p* < 0.001; quadratic: r = 0.430, *p* < 0.001). Third, analyses using only pre-2019 data to avoid the potential effect of COVID-19 also showed a similar pattern, with slightly reduced but still statistically significant correlations (linear: r = 0.269, *p* < 0.001; quadratic: r = 0.283, *p* = 0.002).

#### 3.1.2 Adolescence

**Figure 3:**
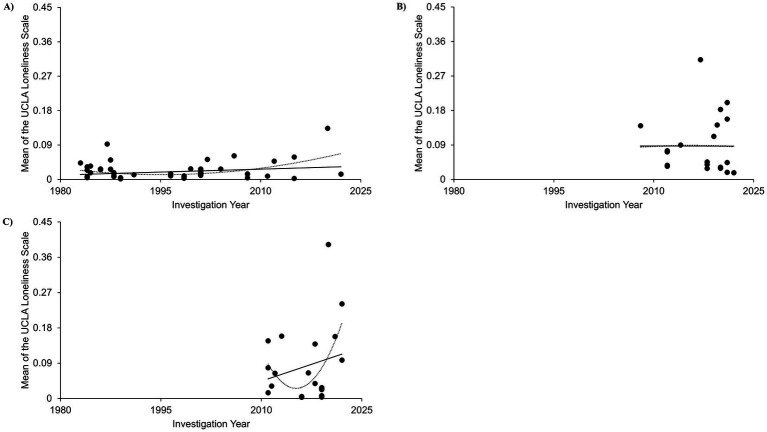
Linear regressions of cross-temporal meta-analysis among developmental stages. **(A)** UCLA loneliness score changes over time among adolescents; *d* = 104, **(B)** UCLA loneliness score changes over time among adults; *d* = 22, and **(C)** UCLA loneliness score changes over time among senium individuals; *d* = 21.

A linear regression analysis on the datasets from adolescent participants indicated a low correlation between loneliness scores and investigation year (r = 0.239, *p* = 0.014) (Figure 3). The quadratic regression results were also significant (r = 0.443, *p* < 0.001).

#### 3.1.3 Adulthood

Results of a linear regression analysis on the datasets from adult participants were not significant (r = 0.001, *p* = 0.998) (Figure 3). The quadratic regression results were not significant either (r = 0.000, *p* = 0.999).

#### 3.1.4 Senium

Results of a linear regression analysis on the datasets from senium participants were not significant (r = 0.224, *p* = 0.331) (Figure 3). The quadratic regression results were not significant either (r = 0.464, *p* = 0.113).

### 3.2 Multiple regression analysis

#### 3.2.1 Overall

A multiple regression analysis was conducted using all of the data (Table 1). R^2^ was 0.34 (*p* < 0.001), explaining approximately 30% of the variance in mean scores. Adulthood and senium had a significant negative effect on the mean score. Additionally, the investigation year had a significant negative effect, indicating that the mean score increased as the investigation year advanced.

**Table 1:** Results of multiple regression analysis of all data.

Variable	*B*	SE *B*	95% CI	β
(Constant)	0.023	0.003	(0.016, 0.029)	
Scale type				
Point scale	−0.005	0.004	(−0.012, 0.002)	−0.116
Number of items	−0.006	0.004	(−0.013, 0.001)	−0.174
Translation (Kudo)	−0.001	0.002	(−0.006, 0.003)	−0.052
Translation (Moroi)	0.003	0.003	(−0.003, 0.008)	0.071
Translation (Toyoshima)	0.003	0.004	(−0.004, 0.010)	0.068
Translation (Masuda)	0.006	0.004	(−0.001, 0.013)	0.136
Translation (Arimoto)	0.008	0.005	(−0.002, 0.018)	0.130
Developmental stage				
Adolescence	−0.004	0.003	(0.010, 0.002)	−0.160
Adulthood	−0.011	0.003	(−0.017, 0.004)	−0.267**
Senium	−0.011	0.003	(−0.017, −0.005)	−0.274***
Investigation year	0.000	0.000	(0.000, 0.000)	−0.276*
*R* ^2^				0.335***

*p < 0.05, **p < 0.01, ***p < 0.001; d = 183; *B*; unstandardized coefficient; CI; confidence interval; β; standardized coefficient.

#### 3.2.2 Adolescence

A multiple regression analysis was conducted using data from adolescent participants (Table 2). The investigation year was centered according to the adolescents’ data. The results indicated that utilizing 20 items negatively affected the mean score. Some translation types positively affected the mean score. The investigation year had a positive effect, indicating that the mean score increased over time.

**Table 2:** Multiple regression analysis results for adolescent participants.

Variable	*B*	SE *B*	95% CI	β
(Constant)	0.025	0.004	(0.017, 0.032)	
Scale type				
Point scale	0.002	0.005	(−0.007, 0.012)	0.073
Number of items	−0.024	0.007	(−0.37, −0.011)	−0.616***
Translation (Kudo)	0.003	0.001	(0.000, 0.005)	0.251*
Translation (Moroi)	0.005	0.001	(0.002, 0.008)	0.360***
Translation (Toyoshima)	0.013	0.005	(0.003, 0.023)	0.239**
Investigation year			(0.000, 0.000)	
*R* ^2^				0.308***

*p* < 0.05, **p < 0.01, ***p < 0.001; d = 104; *B*; unstandardized coefficient; CI; confidence interval; β; standardized coefficient.

#### 3.2.3 Adulthood

A multiple regression analysis was conducted using data from adult participants (Table 3). The results indicated that some translation types positively affected the mean score.

**Table 3:** Multiple regression analysis results for adult participants.

Variable	*B*	SE *B*	95% CI	β
(Constant)	0.000	0.004	(−0.008, 0.009)	
Scale type				
Point scale	0.010	0.005	(0.000, 0.021)	0.434
Number of items	−0.008	0.005	(−0.019, 0.002)	−0.390
Translation (Kudo)	0.030	0.005	(0.019, 0.041)	0.878***
Translation (Moroi)	0.032	0.013	(0.004, 0.060)	0.672*
Translation (Toyoshima)	0.002	0.005	(−0.007, 0.012)	0.099
Translation (Masuda)	0.009	0.006	(−0.004, 0.022)	0.374
Translation (Arimoto)	0.011	0.004	(0.021, 0.440)	0.434*
Investigation year	−0.001	0.001	(0.001, 0.001)	−0.228
*R* ^2^				0.828***

*p < 0.05, ***p < 0.001; d = 22; *B*; unstandardized coefficient; CI; confidence interval; β; standardized coefficient.

#### 3.2.4 Senium

Results of a multiple regression analysis conducted using data from senium participants indicated that the coefficient of determination was not significant (*R*^2^ = 0.371, *p* = 0.290).

### 3.3 Sex difference analysis

**Figure 4:**
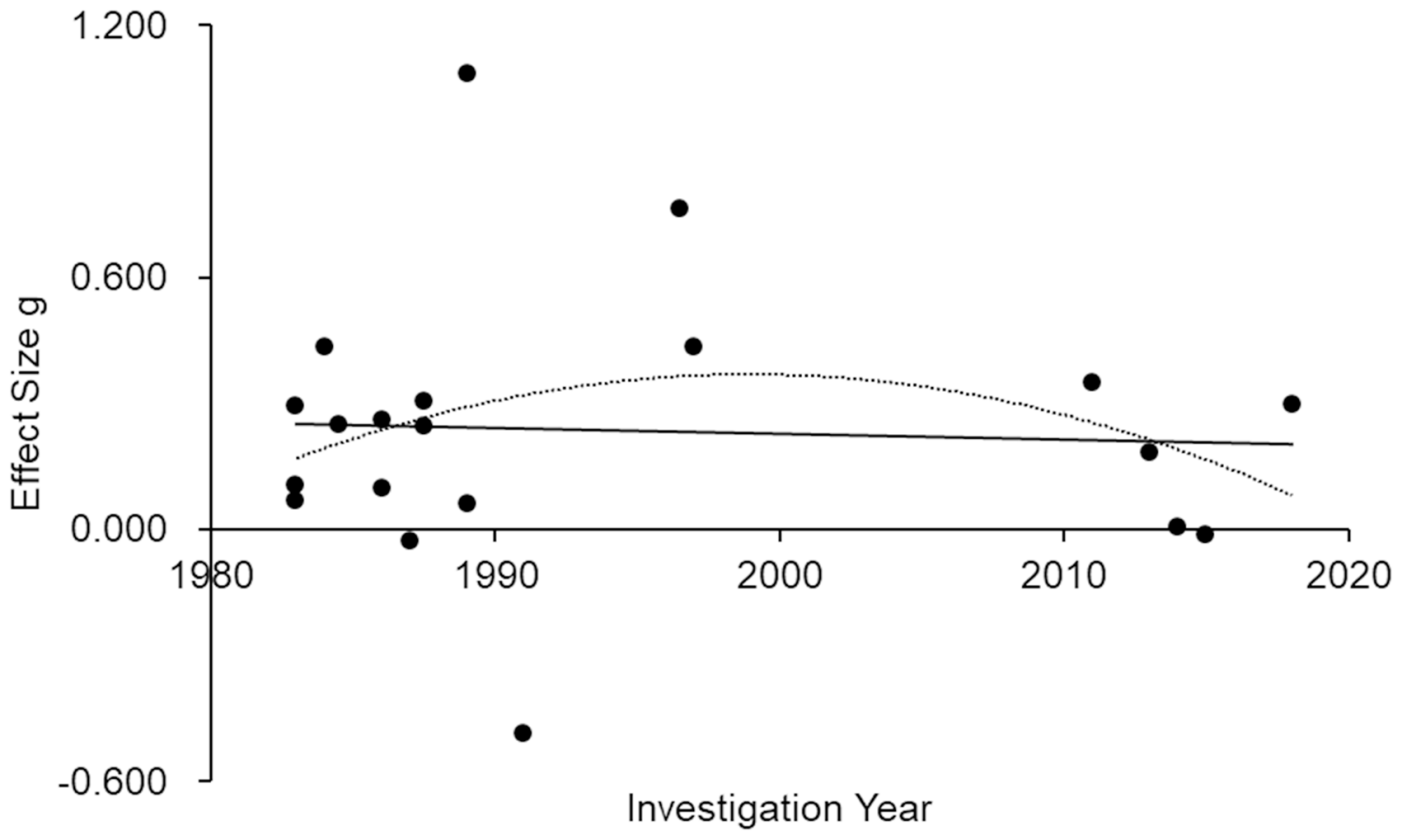
UCLA loneliness score effect size changes over time among male and female participants; *d* = 20.

The effect size between loneliness in male and female participants was 0.19, indicating higher loneliness among the former than among the latter. A single regression analysis was performed to determine the correlation between effect size and investigation year (Figure 4). No significant result was found (r = 0.055, *p* = 0.815). The quadratic regression was not significant either (r = 0.476, *p* = 0.113).

**Figure 5:**
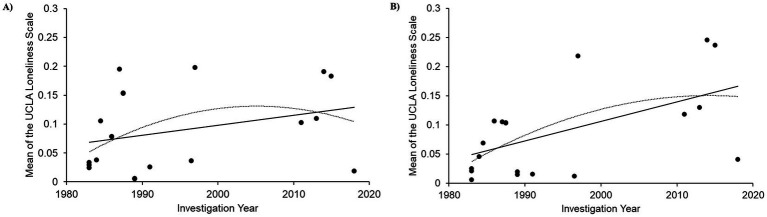
Linear regressions of cross-temporal meta-analysis among male and female participants. **(A)** UCLA loneliness score changes over time among males; *d* = 20, **(B)** UCLA loneliness score changes over time among females; *d* = 20.

The result of a regression analysis on data from male participants in studies using the 20-item scale was not significant (r = 0.312, *p* = 0.181) (Figure 5). The quadratic regression result was not significant either (R^2^ = 0.097, *p* = 0.181). For female participants in studies using the 20-item scale, the linear regression analysis indicated a significant positive correlation (r = 0.552, *p* = 0.012) (Figure 5). The quadratic regression result was also significant (R^2^ = 0.305, *p* = 0.012).

Subsequently, multiple regression analysis was conducted using data from male participants. The result showed that the coefficient of determination was not significant (*R*^2^ = 0.767, *p* = 0.083). Additionally, multiple regression analysis was conducted using data from female participants; the results are presented in Table 4. Kudo translation negatively affected the mean score, while Toyoshima and Arimoto translations positively affected the mean score.

**Table 4:** Multiple regression analysis results for female participants.

Variable	*B*	SE *B*	95% CI	β
(Constant)	0.078	0.034	(−0.004, 0.152)	
Scale type				
Point scale	0.020	0.032	(−0.49, 0.089)	0.109
Translation (Kudo)	−0.059	0.017	(0.095, 0.023)	−0.400***
Translation (Masuda)	0.017	0.036	(−0.061, 0.094)	0.068
Translation (Toyoshima)	0.141	0.052	(0.028, 0.255)	0.576*
Translation (Arimoto)	0.137	0.045	(0.039, 0.236)	0.406*
Developmental stage				
Senium	−0.019	0.031	(−0.87, 0.048)	−0.093
Investigation year	−0.001	0.001	(0.004, 0.002)	−0.176
*R^2^*				0.953***

*p < 0.05, ***p < 0.001; d = 20; *B*; unstandardized coefficient; CI; confidence interval; β; standardized coefficient.

### 3.4 Effect size analysis of the COVID-19 pandemic

The effect size of the COVID-19 pandemic was calculated using 15 datasets from 2017 to 2019 and 7 datasets from 2020 to 2022. The mean score for 2017–2019 was 39.80 and that for 2020–2022 was 43.96. The effect size was −0.263 (95% CI = −0.305, −0.220), indicating a negative effect.

### 3.5 Social indicators

Table 5 presents the correlations between social indicators and loneliness. The results showed that loneliness was significantly correlated with all indicators.

**Table 5:** Correlations between social indicators and loneliness.

Social indicator	10 years prior to data collection	5 years prior to data collection	Actual year	5 years after data collection	10 years after data collection
Social connectedness					
Number of single-person households	0.389***	0.380***	0.460***	0.322***	0.256*
Average household size	−0.434***	−0.406***	−0.407***	−0.292***	−0.208*
Marriage rate (MHLW)	−0.327	−0.314*	−0.294*	−0.171	0.000
Divorce rate (MHLW)	−0.369	−0.198	−0.276*	−0.222	0.070
Marriage rate (MIC)	−0.366***	−0.459***	−0.487***	−0.457***	−0.198*
Divorce rate (MIC)	0.333***	0.209**	−0.042	−0.130	−0.322***
Social interaction within the community	−0.102	−0.339*	−0.224	−0.219	−0.346
Frequent social interactions within the community	−0.423*	−0.233	−0.153	0.030	−0.466*
Economic conditions					
GDP	−0.126	0.204*	0.299***	0.197	0.327***
Total unemployment rate	0.356***	0.151*	−0.108	−0.133	−0.404***
Overall threat					
Average time spent using the internet (work days)	n/a	0.424*	0.287*	0.179	0.099
Average time spent using the internet (holidays)	n/a	0.463**	0.301*	0.174	0.003
Percentage of internet users (work days)	n/a	0.365*	0.191	0.241	−0.061
Percentage of internet users (holidays)	n/a	0.422*	0.184	0.224	−0.076

*p < 0.05, ***p < 0.001; d varies from 11 to 183 because of missing indicators for some years; MHLW – Ministry of Health, Labour and Welfare, MIC – Ministry of Internal Affairs and Communications.

## 4 Discussion

This study found an increase in loneliness in Japanese society between 1983 and 2023. Loneliness also increased among adolescents. The multiple regression analysis indicated that certain developmental stages, the number of items on the scale, and the type of Japanese translation were associated with loneliness. Moreover, loneliness increased among female participants. However, the effect size suggested that male participants consistently reported greater loneliness. All social indicators were significantly associated with loneliness.

By developmental stage, this study found increased loneliness only among adolescents. A previous study also indicated rising loneliness in this group (Twenge et al., 2021). The reasons for adolescents’ loneliness are manifold: peer rejection and victimization, depression, social anxiety, internalizing symptoms, low self-esteem, shyness, and neuroticism (Dahlberg et al., 2022). Depression and lower self-esteem have increased among adolescents in Japan and may partly explain the rise in loneliness. In addition to existing interventions, a new support system is required to address this issue. Further, the multiple regression analysis results indicated that the type of Japanese translation, the number of items, and the investigation year significantly affected loneliness. Although the Japanese versions of the UCLA Loneliness Scale differ slightly in expression, their meanings are consistent with the original English version. However, even small differences in translation may lead to varied interpretations and influence results. Because translation and item variation complicate comparison, a unification of scales is necessary.

The multiple regression analysis of the overall data revealed lower loneliness among adult and senium participants. Further, the latter reported less loneliness compared with adults. Previous studies found that loneliness decreases with age (Barreto et al., 2021; Pyle and Evans, 2018), and the present study’s results suggest a similar developmental pattern in Japan. Although adults and senium participants showed no increase, loneliness remains a concern. Over 20% of adults in a national survey reported feeling lonely, either chronically or occasionally, although this proportion remains lower than among adolescents (Cabinet Office, 2024a). Notably, loneliness remains a social issue in Japanese society, and interventions and support tailored to developmental stages are needed to prevent loneliness.

This study also found increased loneliness among females. However, effect sizes from more studies indicated that males experience greater loneliness. Thus, while males are generally more prone to loneliness, rates among females have increased in recent years. A meta-analysis of 45 countries found no significant sex difference in loneliness (Maes et al., 2019). Therefore, the sex differences in loneliness in Japan may be related to cultural differences. In Japan, self-esteem is higher among men (Oshio et al., 2016), while depression is more prevalent among females [Ministry of Health, Labour and Welfare (MHLW), 2022b].

Furthermore, higher loneliness among females may be due to lifestyle changes, such as increased participation in the workforce (Statistics Bureau of Japan, 2025) and reduced family communication. The multiple regression analysis also indicated that translation type influenced the results. Future studies should use the same UCLA Loneliness Scale to ensure precise longitudinal comparisons.

This study’s results indicated that loneliness increased during the COVID-19 pandemic. A study based on the national survey suggested that individuals who were socially isolated experienced greater loneliness than those who were not (Murayama et al., 2021). Even after social isolation decreased from 2020 to 2021, loneliness increased (Murayama et al., 2021). Conversely, a study in the United States found no increase in loneliness after the pandemic (Luchetti et al., 2020). These findings indicate that social isolation does not always affect loneliness. Even during physical isolation, resilience factors may help mitigate loneliness. However, resilience may function differently in Japan compared with the United States, as individuals are more reluctant to seek professional support in the former (Mojaverian et al., 2013). Consequently, social support may not reach those who need it.

This study also identified correlations between loneliness and various social indicators. However, the correlations should be interpreted carefully, as they are supplementary in nature and do not provide evidence of causal relationships. The number of single-person households was positively correlated with loneliness, with household size and marriage rate being negatively correlated. These results indicate that objective social connectedness and loneliness covary, supporting the validity of this cross-temporal meta-analysis. For these indicators, the data from 10 and 5 years prior to and 10 and 5 years after data collection were significantly correlated with loneliness. Previous research also reported an association between loneliness and these indicators (Greenfield and Russell, 2011; Stack, 1998). These indicators may also reflect the degree of family connectedness, with shifts toward nuclear family structures and non-marital lifestyles potentially weakening family ties, a key form of social connection. Still, whether the increase in single-person households or the decrease in household size and marriage rates causes loneliness remains an open question.

Divorce rates from 10 and 5 years prior were positively correlated with loneliness, suggesting that increases in divorce rates may be related to rises in loneliness. Another study also found an association between divorce and loneliness (Amato, 2000). Higher divorce rates may be associated with cultural shifts toward more flexible and less stable family relationships. However, MHLW data from the actual year and MIC data from 10 years after showed a negative correlation between loneliness and divorce rates; thus, further investigation is necessary.

Social interaction within the community was negatively correlated with loneliness. Saito and Hirano (2019) also reported that lack of contact with neighbors was associated with high loneliness. Lower levels of community contact may reduce opportunities for social connection, thereby increasing feelings of isolation. Once again, data from 10 years after showed similar correlations with loneliness, highlighting the need for further research.

Economic conditions were also associated with loneliness. GDP and loneliness have both been increasing overall, resulting in a positive correlation. Although individuals of lower economic status report higher loneliness (Beller, 2024; Kung et al., 2022), the findings of this study show that the economic abundance of society might not effectively decrease loneliness. One meta-analysis found no association between GDP and loneliness among adolescents (Twenge et al., 2021), suggesting that associations may differ by cultural background or developmental stage. In Japan, higher GDP may be associated with increased working time and lifestyle changes, potentially reducing time available for social connections. The total unemployment rates of 10 and 5 years prior were positively associated with loneliness, suggesting that individuals experiencing job loss also lose social connections, aligning with a previous study that found an association between unemployment and loneliness (Morrish and Medina-Lara, 2021).

Data from 5 years prior and the actual year showed positive correlations between loneliness and Internet use. Greater Internet use may reduce opportunities and motivation for face-to-face communication, thereby decreasing direct social interaction. However, the relationship between Internet usage and loneliness is bidirectional and complex (Bonsaksen et al., 2023; Nowland et al., 2018; Wilson et al., 2012), necessitating further research.

This study has several limitations. First, it is unclear whether lonely individuals participated in the primary studies. Lonely individuals may not be socially isolated (Taylor et al., 2023), and those who feel lonely and are socially isolated may not have been included in the data analyzed. Additionally, individuals who lacked time or were not in the mental space to answer the survey may have been excluded. Therefore, the results do not necessarily reflect the actual loneliness levels of all Japanese citizens. Second, interpretations of the UCLA Loneliness Scale may have changed over time. Given that loneliness and social isolation have been discussed as social issues, individuals may be aware of being lonely and more likely to admit their feelings of isolation. A previous study indicated that the responses on the UCLA Loneliness Scale changed depending on one’s muscularity (Cramer and Neyedley, 1998). How an individual responds to the survey can vary according to their perspective. Therefore, the correlations may reflect a change in individuals’ perspectives rather than a change in loneliness. Third, this study does not reveal the detailed mechanisms of loneliness. By discussing the reasons for the increase in loneliness, the present study listed some of the factors that may be related to loneliness. Future studies should provide a more detailed description of the factors and mechanisms affecting loneliness.

## 5 Conclusion

This study examined changes in loneliness over time in Japanese society and identified related factors using a cross-temporal meta-analysis. In addition, simple and multiple regression analyses, sex difference analysis, an effect size analysis of the COVID-19 pandemic, and correlation analysis of social indicators were conducted.

The results showed that loneliness significantly increased in Japan between 1983 and 2023, particularly among adolescents. Additionally, male individuals were more likely to feel lonely than female individuals; however, loneliness in the female population has increased in recent years. The COVID-19 pandemic was also associated with a further rise in loneliness. Factors associated with loneliness included investigation year, developmental stage, and the version of the UCLA Loneliness Scale used. Social indicators covaried with loneliness over the study period (1983–2023). These findings indicate that loneliness in Japanese society is worsening and that addressing it is an urgent issue. As this study does not explain the precise causes of increased loneliness, further research is needed.

## Data Availability

The original contributions presented in the study are included in the article/Supplementary material, further inquiries can be directed to the corresponding author.
