# Supplementary material for: Increasing loneliness in Japan, 1983–2023: a cross-temporal meta-analysis
Source: Front Psychol. 2026 Apr 14;17:1824941. doi: 10.3389/fpsyg.2026.1824941 (PMC13121347; doi:10.3389/fpsyg.2026.1824941)
Supplement: Supplementary file 1 [file Data_Sheet_1.docx]

**Increasing Loneliness in Japan, 1983–2023: A Cross-Temporal Meta-Analysis**

SPSS analysis code

1. **Single regression analysis**

All data, each developmental stage, male/female:

CURVEFIT

/VARIABLES=Loneliness_Score WITH Investigation_Year

/CONSTANT

/MODEL=LINEAR QUADRATIC

/PLOT FIT.

Sex difference:

CURVEFIT

/VARIABLES=Effect_Size WITH Investigation_Year

/CONSTANT

/MODEL=LINEAR QUADRATIC

/PLOT FIT.

1. **Multiple regression analysis**

All data:

REGRESSION

/DESCRIPTIVES MEAN STDDEV CORR SIG N

/MISSING LISTWISE

/STATISTICS COEFF OUTS CI(95) R ANOVA CHANGE

/CRITERIA=PIN(.05) POUT(.10) TOLERANCE(.0001)

/NOORIGIN

/DEPENDENT Loneliness_Score

/METHOD=ENTER Investigation_Year Adolescence Adulthood Senium Point_Scale Number_of_Items

Translation_Kudo Translation_Moroi Translation_Toyoshima Translation_Masuda Translation_Arimoto.

Each developmental stage:

REGRESSION

/DESCRIPTIVES MEAN STDDEV CORR SIG N

/MISSING LISTWISE

/STATISTICS COEFF OUTS CI(95) R ANOVA CHANGE

/CRITERIA=PIN(.05) POUT(.10) TOLERANCE(.0001)

/NOORIGIN

/DEPENDENT Loneliness_Score

/METHOD=ENTER Point_Scale Number_of_Items Translation_Kudo Translation_Moroi

Translation_Toyoshima Translation_Masuda Translation_Arimoto Investigation_Year.

1. **Social indicators**

CORRELATIONS

/VARIABLES=Social_Indicator Loneliness_Score

/PRINT=TWOTAIL NOSIG FULL

/STATISTICS DESCRIPTIVES

/MISSING=PAIRWISE.
