# Supplementary material for: Increasing loneliness in Japan, 1983–2023: a cross-temporal meta-analysis
Source: Front Psychol. 2026 Apr 14;17:1824941. doi: 10.3389/fpsyg.2026.1824941 (PMC13121347; doi:10.3389/fpsyg.2026.1824941)
Supplement: Supplementary file 2 [file Data_Sheet_2.docx]

**Increasing Loneliness in Japan, 1983–2023: A Cross-Temporal Meta-Analysis**

Strategy for Coding and Score Adjustments

The following were coded from each study: mean UCLA loneliness score, standard deviation, sample size, investigation year, point scale, number of items on the scale, type of Japanese translation of the scale, and age/school level or working status/developmental stage (Supplemental Table 1). In studies involving experimental manipulations, pretest, rather than posttest, means were recorded. To analyze by generation, age/school level or working status/developmental stage was converted into developmental stages (i.e., childhood, adolescence, adulthood, and senium). Childhood, adolescence, adulthood, and senium included, respectively, elementary school students, junior high school and university students, participants who were working and not attending school, and individuals aged ≥65 years. If both age and school level/working status were reported in a study, school level/working status was used. Data involving two or more developmental stages were excluded from the analysis. Because of the paucity of studies on childhood, this developmental stage was not analyzed.

Various methods were used to determine the UCLA loneliness score. Therefore, some adjustments were required before conducting the analysis. First, the score range was set to begin from 1. Second, because the number of items on the scale varied, the mean score per item was calculated. Third, because point scales differed, the mean scores were adjusted to a 4-point scale. To make this adjustment, the deviation was multiplied by 3 (the range of a 4-point scale), divided by the range of the original point scale, and then 2.5 (the median of a 4-point scale) was added. Finally, all mean scores were weighted by sample size: each sample size was multiplied by its adjusted average score and divided by the total of all sample sizes. The inverse variance method could not be used because of the adjustments made to the scores.
